# Late-Onset Ovarian Bleeding After Transvaginal Oocyte Retrieval in a Patient With Suspected Hereditary Hemorrhagic Telangiectasia

**DOI:** 10.7759/cureus.45657

**Published:** 2023-09-21

**Authors:** Kohei Tamura, Hironori Takahashi, Suzuki Tatsuya, Mai Ohhashi, Hiroyuki Fujiwara

**Affiliations:** 1 Obstetrics and Gynecology, Jichi Medical University, Shimotsuke, JPN; 2 Reproductive Medicine Center, Dokkyo Medical University Hospital, Mibu, JPN

**Keywords:** transvaginal oocyte retrieval, ovarian bleeding, inclusion cyst, hereditary hemorrhagic telangiectasia, hemoperitoneum

## Abstract

Assisted reproductive technology (ART) requires transvaginal oocyte retrieval (TVOR), and ovarian bleeding after TVOR rarely occurs. We present a case of a 37-year-old woman (0-gravida) who was diagnosed with possible hemorrhagic telangiectasia (HHT) and had a history of three laparotomies for ovarian bleeding and an inclusion cyst adjacent to the right ovary after the third operation. HHT is a hereditary disease characterized by spontaneous hemorrhage of some organs, such as the nose, brain, lungs, gastrointestinal tract, and liver. She desired ART after fertility treatment and then had abdominal pain with ovarian swelling five days after TVOR. Moreover, both the right ovary and inclusion cyst were gradually swollen with hematoma. Finally, abdominal pain and the hemoglobin level deteriorated, necessitating an emergency surgery on the eighth day. We notify reproductive physicians that patients with HHT may readily develop ovarian bleeding with or without inclusion cysts after TVOR, although inclusion cysts may also be associated with late-onset bleeding.

## Introduction

Hereditary hemorrhagic telangiectasia (HHT) is characterized by autosomal dominant inheritance, recurrent nasal bleeding, visceral arteriovenous malformation (AVM), and peripheral vasodilatation of skin mucosa [1]. The incidence of HHT is 0.02% [2]. Pregnancies associated with assisted reproductive technology (ART) have been increasing in developed countries. ART requires transvaginal oocyte retrieval (TVOR), and TVOR can induce bleeding, infection, and anesthesia-related side effects [3,4]. Of those, peritoneal bleeding involving ovarian bleeding occurs after 0.23% of TVOR procedures [5]. In a systematic review, severe hemoperitoneum resulting from ovarian bleeding related to TVOR occurred in 0.08% (23/28416). Regarding the timing between TVOR and the onset of bleeding-related symptoms, the majority emerged within 24 hours following TVOR [6]. Indeed, 93.3% (28/30) of the patients who underwent surgical intervention showed symptoms within 24 hours after TVOR [6]. Here, we present a woman with suspected HHT, who showed late-onset severe ovarian bleeding, requiring an emergency hemostatic surgery on the eighth day following TVOR.

## Case presentation

**Figure 1:**
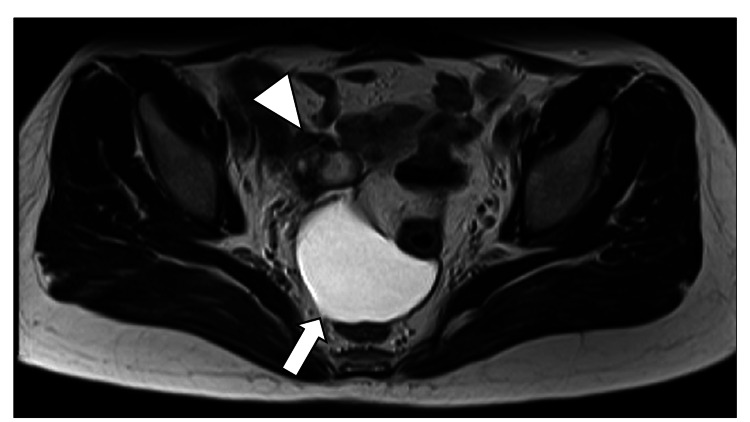
Magnetic resonance imaging of the inclusion cyst (T2-weighted) before transvaginal oocyte retrieval. An inclusion cyst (arrow) adjacent to the right ovary (arrowhead) appeared.

A 37-year-old woman (0-gravida) had undergone ART in an attempt to conceive. Her mother was affected by HHT. She was not diagnosed with definite HHT; however, she sometimes had nasal bleeding and headaches. In addition, emergent hemostatic surgeries were performed three times for ovarian bleeding and twice for subdural hematoma. She had an inclusion cyst around the right ovary after the third laparotomy for ovarian bleeding at 23 years old (Figure 1).

**Figure 2:**
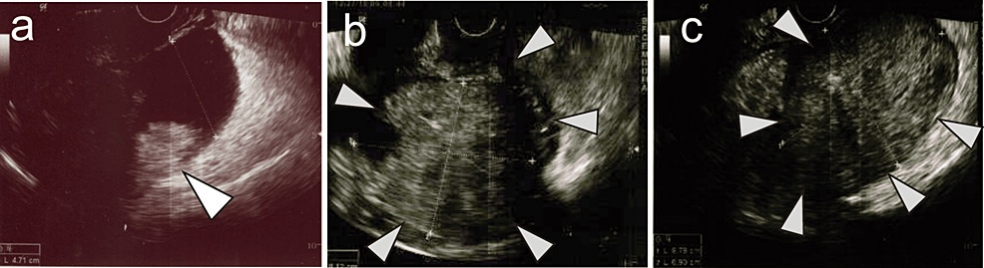
Transvaginal ultrasound findings after transvaginal oocyte retrieval (TVOR). (A) Hematoma was suspected in the inclusion cyst by ultrasound six days after TVOR (arrowhead). (B) Swollen ovary (arrowhead) was observed eight days after TVOR. (C) An inclusion cyst filled with hematoma was detected eight days after TVOR (arrowhead).

Taken together, she was highly suspected of HHT. Slight ovarian bleeding repeatedly occurred at every ovulation based on transvaginal ultrasound. Sex hormone levels, the cervical mucus test, and semen examination showed no abnormalities. Intrauterine inseminations (IUI) ended in the second failure at 35 years old. Considering the insufficient diffusion of hysterosalpingography, the treatments changed from IUI to ART despite the risk of ovarian bleeding. Adopting the gonadotropin-releasing hormone (GnRH) agonist short protocol, we performed TVOR with a fine needle (17 G) and 180-mmHg suction pressure to two follicles from the right ovary. We conducted it carefully with color Doppler mode transvaginal ultrasound and collected two oocytes. Transvaginal ultrasound showed no sign of fluid in the peritoneal cavity two hours after TVOR. However, she visited the emergency room due to abdominal pain five days after TVOR. A swollen right ovary (6.6 x 5.6 cm) and an inclusion cyst on the right ovary were noted, and, thus, we suspected ovarian bleeding. The hemoglobin level was 14.7 g/dL, blood pressure was 122/94 mmHg, and pulse was 64 beats/minute. We observed her course naturally. On the following day (six days after TVOR), the right ovary was larger, and high echogenicity in the inclusion cyst had also changed, suggesting hemoperitoneum associated with ovarian bleeding (Figure 2A). Her vital signs remained stable, and we continued to observe her course cautiously. Two days later (eight days after TVOR), the abdominal pain had intensified, and the right ovary including hematoma had increased in size (8.1 x 6.9 cm) (Figure 2B). The size of the inclusion cyst (8.8 x 6.9 cm) (Figure 2C) was also larger. The hemoglobin level decreased to 9.3 g/dL. An emergency laparotomy was performed. The intra-abdominal cavity showed that the right ovary was swollen with hematoma and the inclusion cyst was filled with blood including coagulation. Intraoperative bleeding was 1600 mL. She was transfused with six units of red blood cells and two units of fresh frozen plasma. The postoperative course was uneventful, and she left the hospital seven days following the emergent surgery. She discontinued her infertility treatment. Informed consent for reporting was obtained from the patient.

## Discussion

There are a few important clinical findings in this report. First, the patient with suspected HHT showed severe ovarian bleeding requiring hemostatic surgery several days after TVOR. To the best of our knowledge, this is the first report on ovarian bleeding associated with TVOR in a patient suffering from suspected HHT. Second, the manifestation of bleeding can be delayed by an inclusion cyst.

A patient with HHT exhibited severe ovarian bleeding requiring hemostatic surgery after TVOR. Several reports showed that patients with HHT are prone to bleeding in other organs [7,8]. Patients with HHT have mucous telangiectasia and AVM that can affect the nose, brain, lungs, gastrointestinal tract, and liver [7]. Recurrent nasal bleeding and gastrointestinal bleeding occur in approximately 90% [8] and 20% [7] of patients, respectively. Although there is no report on ovarian bleeding in patients with HHT, the bleeding tendency may also occur in ovaries. There is also no report on fertility treatment involving a patient with HHT. The patient required multiple laparotomies associated with ovarian bleeding. We considered that this patient had fragile vessels, including AVM in her ovary. Although similar additional cases need to be accumulated, it may be necessary to conduct TVOR in patients with HHT with caution.

**Figure 3:**
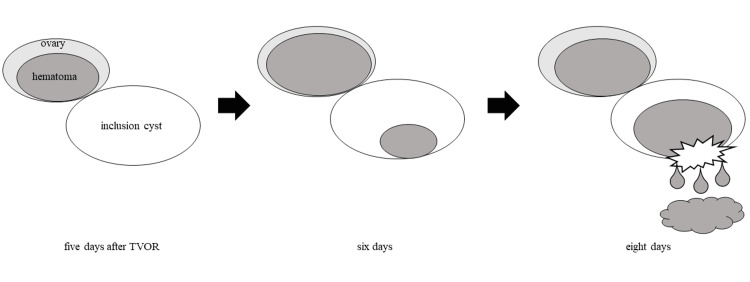
Schema of late-onset ovarian bleeding. Ovarian hematoma was detected five days after transvaginal oocyte retrieval (TVOR), and it gradually enlarged. Bleeding started to flow into the inclusion cyst six days after TVOR. The cyst ruptured, leading to bleeding into the intraperitoneal cavity eight days after TVOR. Image Credits: Kohei Tamura.

Inclusion cysts adjacent to ovaries may be associated with late-onset ovarian bleeding. A previous study reported that initial signs of hemoperitoneum after TVOR were evident in more than 90% within 24 hours [6]. Significant bleeding occurred five days after TVOR in this case. Patients with HHT have abnormal fragile vessels including AVM, and, thus, vessels may be easily damaged by puncture. In addition, puncture can cause inflammation promoted by injury and infection [9]. We considered the following scenario (Figure 3). Ovarian bleeding flowed into the inclusion cyst soon after TVOR and then it started to leak into the intraperitoneal cavity several days after TVOR. The new bleeding was triggered by the leakage. Another report supports our hypothesis, describing how the rupture of pancreatic pseudocysts containing blood triggered new intraperitoneal bleeding [10]. Although we do not know whether late-onset bleeding is due to HHT, an inclusion cyst, or both, clinicians should be aware of the possibility that these patients are vulnerable to delayed bleeding after TVOR.

A previous study reported that risk factors for ovarian bleeding after TVOR were low body mass index, young age, high number and long duration of oocyte retrieval, inexperienced surgeon, history of laparotomy, and presence of pelvic inflammatory disease in 23,000 TVOR cases [5]. In our case, the history of laparotomy was applicable to the risk factors, and we think it might partially contribute to severe ovarian bleeding because inflammation of surgery made a bad impact on ovarian vessels to be vulnerable to irritations. Considering these factors, the risk of ovarian bleeding should be reduced by performing the following strategies in a patient with HHT. First, a low-dosage pill to inhibit ovulation should be employed to prevent ovarian bleeding. Second, reproductive physicians should modulate follicle stimulation and decrease the number of aspirations. Third, preventive hemostatic agents, including carbazochrome sodium sulfonate and tranexamic acid, should be prescribed after TVOR. Moreover, some studies reported that the intravenous dosage of bevacizumab markedly improved hemoglobin and decreased the frequency of red blood cell transfusion in HHT patients with hepatic AVM [11] and a number of episodes of nasal bleeding [12]. Therefore, this drug may be one of the options to prevent or improve ovarian bleeding.

## Conclusions

Patients with HHT may easily develop ovarian bleeding as well as bleeding in other organs such as those described above for fragile vessels, which are susceptible to various stimuli. We are not sure whether inclusion cysts are also associated with late-onset bleeding; however, it is likely that bleeding flowed in the cyst after TVOR and then it was full and ruptured. Furthermore, bleeding in the cysts leaked into the intraperitoneal cavity for several days.

It is the key point in this report that reproductive physicians should recognize that patients with HHT may be at a high risk of ovarian bleeding with or without the inclusion cyst after TVOR. Further studies are required to clarify the phenomenon, and asking patients suspected or definite with HHT to visit the hospital for several days after TVOR in order not to miss the possibility of severe ovarian bleeding may be one of the solutions for revealing it.

## References

[1] Shovlin CL, Guttmacher AE, Buscarini E (2000). Diagnostic criteria for hereditary hemorrhagic telangiectasia (Rendu-Osler-Weber syndrome). Am J Med Genet.

[2] Dakeishi M, Shioya T, Wada Y (2002). Genetic epidemiology of hereditary hemorrhagic telangiectasia in a local community in the northern part of Japan. Hum Mutat.

[3] Aragona C, Mohamed MA, Espinola MS, Linari A, Pecorini F, Micara G, Sbracia M (2011). Clinical complications after transvaginal oocyte retrieval in 7,098 IVF cycles. Fertil Steril.

[4] Ludwig AK, Glawatz M, Griesinger G, Diedrich K, Ludwig M (2006). Perioperative and post-operative complications of transvaginal ultrasound-guided oocyte retrieval: prospective study of >1000 oocyte retrievals. Hum Reprod.

[5] Levi-Setti PE, Cirillo F, Scolaro V (2018). Appraisal of clinical complications after 23,827 oocyte retrievals in a large assisted reproductive technology program. Fertil Steril.

[6] Nouri K, Walch K, Promberger R, Kurz C, Tempfer CB, Ott J (2014). Severe haematoperitoneum caused by ovarian bleeding after transvaginal oocyte retrieval: a retrospective analysis and systematic literature review. Reprod Biomed Online.

[7] Kritharis A, Al-Samkari H, Kuter DJ (2018). Hereditary hemorrhagic telangiectasia: diagnosis and management from the hematologist's perspective. Haematologica.

[8] AAssar OS, Friedman CM, White RI Jr (1991). The natural history of epistaxis in hereditary hemorrhagic telangiectasia. Laryngoscope.

[9] Lee HN, Surh YJ (2012). Therapeutic potential of resolvins in the prevention and treatment of inflammatory disorders. Biochem Pharmacol.

[10] Dardik I, Dardik H (1968). Patterns of hemorrhage into pancreatic pseudocysts. Am J Surg.

[11] Flieger D, Hainke S, Fischbach W (2006). Dramatic improvement in hereditary hemorrhagic telangiectasia after treatment with the vascular endothelial growth factor (VEGF) antagonist bevacizumab. Ann Hematol.

[12] Dupuis-Girod S, Ginon I, Saurin JC (2012). Bevacizumab in patients with hereditary hemorrhagic telangiectasia and severe hepatic vascular malformations and high cardiac output. JAMA.

